# Tracheostomy Timing and Outcome in Severe COVID-19: The WeanTrach Multicenter Study

**DOI:** 10.3390/jcm10122651

**Published:** 2021-06-16

**Authors:** Denise Battaglini, Francesco Missale, Irene Schiavetti, Marta Filauro, Francesca Iannuzzi, Alessandro Ascoli, Alberto Bertazzoli, Federico Pascucci, Salvatore Grasso, Francesco Murgolo, Simone Binda, Davide Maraggia, Giorgia Montrucchio, Gabriele Sales, Giuseppe Pascarella, Felice Eugenio Agrò, Gaia Faccio, Sandra Ferraris, Savino Spadaro, Giulia Falò, Nadia Mereto, Alessandro Uva, Jessica Giuseppina Maugeri, Bellissima Agrippino, Maria Vargas, Giuseppe Servillo, Chiara Robba, Lorenzo Ball, Francesco Mora, Alessio Signori, Antoni Torres, Daniele Roberto Giacobbe, Antonio Vena, Matteo Bassetti, Giorgio Peretti, Patricia R. M. Rocco, Paolo Pelosi

**Affiliations:** 1 Anesthesia and Intensive Care, San Martino Policlinico Hospital, IRCCS for Oncology and Neuroscience, 16132 Genoa, Italy; francesca.iannuzzi21@gmail.com (F.I.); kiarobba@gmail.com (C.R.); lorenzo.ball@unige.it (L.B.); ppelosi@hotmail.com (P.P.); 2 Department of Medicine, University of Barcelona, 08007 Barcelona, Spain; 3 Department of Otorhinolaryngology-Head and Neck Surgery, University of Genoa, San Martino Policlinico Hospital, IRCCS for Oncology and Neuroscience, 16132 Genoa, Italy; missale.francesco@gmail.com (F.M.); mfilauro@yahoo.com (M.F.); alessandro.ascoli@outlook.it (A.A.); Francesco.mora@hsanmartino.it (F.M.); Giorgio.peretti@hsanmartino.it (G.P.); 4 Department of Health Sciences, Section of Biostatistics, University of Genoa, 16132 Genoa, Italy; irene.schiavetti@gmail.com (I.S.); alessio.signori@medicina.unige.it (A.S.); 5 Department of Surgical Sciences and Integrated Diagnostics (DISC), University of Genoa, 16132 Genoa, Italy; 6 First Division of Anesthesiology and Intensive Care Unit, ASST Spedali Civili di Brescia, 25123 Brescia, Italy; albertobertazzoli@hotmail.com (A.B.); riauno@spedalicivili.brescia.it (F.P.); 7 Dipartimento dell’Emergenza e Trapianti d’Organo (DETO), Sezione di Anestesiologia e Rianimazione, Università degli Studi di Bari “Aldo Moro”, Ospedale Policlinico, 70124 Bari, Italy; salvatore.grasso@uniba.it (S.G.); francesco.murgolo@uniba.it (F.M.); 8 Anaesthesia and Intensive Care Department, University Hospital, Ospedale di Circolo, 21100 Varese, Italy; Simone.Binda@asst-settelaghi.it (S.B.); medici.terapiaintensiva@ospedale.varese.it (D.M.); 9 Anestesia e Rianimazione 1U, Department of Anesthesia, Intensive Care and Emergency, Città della Salute e della Scienza Hospital, 10121 Turin, Italy; g.montrucchio@gmail.com (G.M.); gabriele.sales86@gmail.com (G.S.); 10 Department of Anaesthesia, Intensive Care and Pain Management, Universita Campus Bio-Medico di Roma, Via Alvaro del Portillo 21, 00128 Rome, Italy; g.pascarella@unicampus.it (G.P.); F.Agro@unicampus.it (F.E.A.); 11 U.O. di Anestesia e Rianimazione, Ospedale di Treviglio-Caravaggio, 24047 Treviglio, Italy; gaiaf88@gmail.com (G.F.); bubasandra@libero.it (S.F.); 12 Department of Morphology, Surgery and Experimental Medicine, Faculty of Medicine, University of Ferrara, 44121 Ferrara, Italy; savinospadaro@gmail.com (S.S.); giulia.falo@edu.unife.it (G.F.); 13 Anestesia e Rianimazione, Ospedale Villa Scassi, 16132 Genoa, Italy; Nadia.Mereto@asl3.liguria.it (N.M.); uva.alessandro87@gmail.com (A.U.); 14 Anesthesia and Intensive Care, “Garibaldi Centro” Hospital, ARNAS Garibaldi, 95100 Catania, Italy; jessicamaugeri@hotmail.it (J.G.M.); dottorenuccio@hotmail.com (B.A.); 15 Dipartimento di Neuroscienze, Scienze Riproduttive e Odontostomatologiche, Università degli Studi di Napoli Federico II, 80126 Napoli, Italy; maria.vargas@unina.it (M.V.); servillo@unina.it (G.S.); 16 Department of Pulmonology, Hospital Clinic of Barcelona, Institut d’Investigacions Biomèdiques August Pi i Sunyer (IDIBAPS), University of Barcelona, SGR 911-Ciber de Enfermedades Respiratorias (CIBERES), 08007 Barcelona, Spain; atorres@clinic.cat; 17 Dipartimento di Scienze della Salute (DISSAL), Università degli Studi di Genova, 16132 Genova, Italy; daniele.roberto.giacobbe@gmail.com (D.R.G.); matteo.bassetti@hsanmartino.it (M.B.); 18 Clinica Malattie Infettive, Istituto di Ricovero e Cura a Carattere Scientifico (IRCCS) per l’Oncologia e le Neuroscienze, 16132 Genova, Italy; anton.vena@gmail.com; 19 Laboratory of Pulmonary Investigation, Carlos Chagas Filho Institute of Biophysics, Federal University of Rio de Janeiro, Rio de Janeiro 21941, Brazil; prmrocco@gmail.com; 20 COVID-19 Virus Network (RedeVírus MCTI), Ministry of Science, Technology, and Innovation, Brasília 70007, Brazil

**Keywords:** tracheostomy, COVID-19, SARS-CoV-2, intensive care, coronavirus, surgical technique, percutaneous

## Abstract

Background: Tracheostomy can be performed safely in patients with coronavirus disease 2019 (COVID-19). However, little is known about the optimal timing, effects on outcome, and complications. Methods: A multicenter, retrospective, observational study. This study included 153 tracheostomized COVID-19 patients from 11 intensive care units (ICUs). The primary endpoint was the median time to tracheostomy in critically ill COVID-19 patients. Secondary endpoints were survival rate, length of ICU stay, and post-tracheostomy complications, stratified by tracheostomy timing (early versus late) and technique (surgical versus percutaneous). Results: The median time to tracheostomy was 15 (1–64) days. There was no significant difference in survival between critically ill COVID-19 patients who received tracheostomy before versus after day 15, nor between surgical and percutaneous techniques. ICU length of stay was shorter with early compared to late tracheostomy (*p* < 0.001) and percutaneous compared to surgical tracheostomy (*p* = 0.050). The rate of lower respiratory tract infections was higher with surgical versus percutaneous technique (*p* = 0.007). Conclusions: Among critically ill patients with COVID-19, neither early nor percutaneous tracheostomy improved outcomes, but did shorten ICU stay. Infectious complications were less frequent with percutaneous than surgical tracheostomy.

## 1. Introduction

The novel coronavirus disease 2019 (COVID-19) is caused by the severe acute respiratory syndrome coronavirus 2 (SARS-CoV-2). As a result of its highly infectious nature and rapid spread, COVID-19 is ravaging health systems worldwide [1,2,3]. Although the majority of individuals experience mild symptoms, the sheer volume of cases caused by the COVID-19 pandemic has led to an incredibly high number of critically ill patients requiring long-term intubation and invasive mechanical ventilation (MV), with prolonged intensive care unit (ICU) stay and sedation requirements [4].

Before the onset of the COVID-19 pandemic, it was common practice in the ICU setting to stratify time to tracheostomy based on distinct definitions of “early” and “late” [5,6,7,8]. Time to tracheostomy is influenced by several factors, including patient and family expectations, predicted outcomes, and the likelihood of weaning from mechanical ventilation [9]. Early tracheostomy was considered helpful in shortening the duration of MV, with the advantage of increasing patient comfort while reducing sedation requirements [10]. However, there is little evidence to support that early versus late tracheostomy improves survival, shortens the duration of mechanical ventilation and ICU length of stay or reduces lower respiratory tract infections [9,10,11,12,13] in general ICU patients.

Similar considerations can be made for surgical versus percutaneous tracheostomy techniques. A randomized controlled trial in general ICU patients investigating differences between the two and their effects on outcome and short-long term complications found a higher rate of systemic infectious complications in the surgical group compared to the percutaneous group, but the difference did not reach statistical significance [14]. Similar rates of ventilator-associated pneumonia with surgical and percutaneous techniques have been reported [11]. In short, to date, there is no evidence to support a definitive recommendation of one technique over the other.

In COVID-19 cases, the possible complications derived from prolonged endotracheal intubation should be weighed against the risk of viral exposure of hospital staff during aerosol-generating procedures [15]. The high rate of failed weaning from the ventilator and failed endotracheal extubation in COVID-19 suggests a potential need for early tracheostomy [16], since it may help shorten the weaning phase and reduce the rate of associated complications [4,17]. Recent guidelines on the management of tracheostomy in COVID-19 recommend using surgical instead of percutaneous techniques to limit aerosol and droplet exposure, although limited data are available on the comparison of these techniques in the COVID-19 population [18,19].

Based on the foregoing, the primary endpoint of this study was the median time to tracheostomy in critically ill COVID-19 patients. Secondary endpoints were the comparative impacts of early versus late tracheostomy and surgical versus percutaneous tracheostomy on survival, length of ICU stay, and post-tracheostomy complications. Several patient characteristics of interest were also assessed, including weaning attempts before tracheostomy and ventilatory settings on days 1, 7, 15, and the day of tracheostomy.

## 2. Materials and Methods

### 2.1. Study Design

This was an observational, retrospective, multicenter study conducted in 11 Italian ICUs (Supplementary Materials, SM) coordinated by the Department of Anesthesia and Intensive Care, San Martino Policlinico Hospital, IRCCS for Oncology and Neuroscience, Genoa, Italy.

This study was reviewed and approved by the relevant Institutional Review Board (Comitato Etico Regione Liguria, Italy, N. 163/2020; San Martino Policlinico Hospital, IRCCS for Oncology and Neuroscience, Genoa, Italy). Written informed consent was waived due to the retrospective nature of the study. This report follows the STrengthening the Reporting of OBservational studies in Epidemiology (STROBE) recommendations.

### 2.2. Patient Selection, Inclusion, and Exclusion Criteria

WeanTrach enrolled critically ill patients with a positive real-time polymerase chain reaction (RT-PCR) nasopharyngeal swab for severe acute respiratory syndrome coronavirus-2 (SARS-CoV-2), admitted to the study ICUs, who underwent surgical or percutaneous tracheostomy.

We excluded patients who did not receive tracheostomy and those aged <18 years. Patients were included between 20 April and 30 June 2020.

Percutaneous dilatational tracheostomies were primarily performed at the bedside in the ICUs by intensivists, whereas the specialist head and neck surgeons performed surgical tracheostomies in patients with difficult anatomy or other presumed obstacles to percutaneous technique. Open tracheostomies were mostly performed in the operating theater, and occasionally at the bedside in the ICU as necessary.

### 2.3. Data Collection

Data were collected from electronic medical records as part of routine ICU care. Access to the data was managed by the coordinating center.

Data of interest included demographic and past medical history from the day of hospital admission to the day of hospital discharge. The following demographic information was collected: age in years, gender, body mass index (BMI), history of hypertension, diabetes mellitus, chronic respiratory disease (defined as chronic obstructive pulmonary disease or asthma), chronic renal disease (defined as estimated glomerular filtration rate <30 mL/min/1.73 m^2^), chronic liver disease (defined as acute or chronic compensated or decompensated liver cirrhosis), chronic heart disease (defined as a history of current or previous cardiac dysfunction), cancer or hematological malignancy, smoking (defined as a current or former smoker), and chronic neurologic disease (any disease of the brain, spinal cord, peripheral neuropathy, cranial nerve disorder, autonomic nervous system disorder, seizure disorder, movement disorder, migraine, central neuropathy, neuropsychiatric illness).

Respiratory settings were collected for both invasively and non-invasively ventilated patients. Parameters included type of ventilation [conventional oxygen therapy (COT), non-invasive mechanical ventilation (NIMV), invasive mechanical ventilation (IMV)], type of IMV (pressure-controlled ventilation, volume-controlled ventilation, assisted ventilation), ventilator settings [tidal volume, respiratory rate, plateau pressure, minute ventilation, positive end-expiratory pressure (PEEP), fraction of inspired oxygen (FiO_2_)], and blood gas parameters [partial pressure of oxygen (PaO_2_), partial pressure of carbon dioxide (PaCO_2_), pHa, PaO_2_/FiO_2_ ratio, bicarbonates, lactates], recorded at days 1, 7, 15, and on the day of tracheostomy.

Time to tracheostomy, time to weaning, time to extubation, time to ICU admission, time from symptom onset to hospital admission, time to endotracheal intubation, the reason for tracheostomy, and date of ICU discharge were collected.

In-ICU events of interest included duration of mechanical ventilation, ICU length of stay, post-tracheostomy ICU length of stay, complications derived from surgical or percutaneous tracheostomy techniques [including hemorrhage, tracheostomy stoma infection, lower respiratory tract infection (defined as positive bronchoalveolar lavage fluid), stenosis, thrombosis, death], the reason for death, and microorganisms found in bronchoalveolar lavage fluid (BALF) before and after tracheostomy.

### 2.4. Definitions

○ Early tracheostomy was defined as a tracheostomy performed within the first 15 days after endotracheal intubation, while late tracheostomy was defined as a tracheostomy performed ≥ 15 days after endotracheal intubation. The reason for selecting 15 days as the cut-off for early and late tracheostomy was based on the median time to tracheostomy performance in our cohort.
○ Time to tracheostomy was defined as the time elapsed from endotracheal intubation to tracheostomy.
○ Time to weaning was defined as the time from endotracheal intubation to the first spontaneous breathing trial (SBT).
○ SBT was defined as the first attempt to reduce respiratory support before extubation (removal of endotracheal tube and respiratory support). The execution of a SBT did not automatically lead to extubation.
○ Time to extubation was defined as the time between the insertion and the removal of an artificial airway such as an endotracheal tube or tracheostomy tube.
○ Extubation was defined as the removal of an artificial airway. This term was used either for the removal of an endotracheal tube or tracheostomy tube.
○ Time to ICU admission was defined as the time from hospital admission to ICU admission.
○ Time to endotracheal intubation in ICU was defined as the time from ICU admission to endotracheal intubation.
○ ICU discharge was defined as the last day of ICU stay, irrespective of death or discharge to another non-ICU ward.
○ Length of ICU stay was defined as the time between ICU admission and ICU discharge, irrespective of death or discharge to another non-ICU ward.
○ Post-tracheostomy ICU length of stay was considered as the time between tracheostomy and ICU discharge.
○ Reasons for tracheostomy were categorized as follows: prolonged weaning expected, neurological impairment (inability to maintain patient airways because of neurological condition), extubation failure (failure of SBT and/or extubation needing re-intubation), and airway failure (inability to maintain patient airways because of upper airway causes).

### 2.5. Statistical Analysis

No sample size calculation was performed because of the exploratory, descriptive, and retrospective nature of this study. For descriptive summary statistics, variables were reported as mean (standard deviation), median (range), or absolute and relative frequencies, as appropriate.

The Mann–Whitney *U* test was applied to evaluate differences in ICU length of stay between groups (surgical versus percutaneous tracheostomy, late versus early timing). The estimation of any association between complications of tracheostomy and type of intervention (surgical versus percutaneous) was assessed with a preliminary univariate analysis (chi-square test with Yates correction or Fisher’s exact test), followed by a multivariate logistic regression model adjusted for all the baseline variables. The same approach was used with tracheostomy timing (early versus late) as a dependent variable. Time to survival was analyzed with Kaplan–Meier estimates; the log-rank test was used for comparison between groups.

Two-sided *p*-values ≤ 0.05 were considered statistically significant. All analyses were performed using SPSS^®^ version 25.0 (IBM Corp., Armonk, NY, USA).

## 3. Results

Overall characteristics at hospital and ICU admission of the 153 tracheostomized COVID-19 patients who fulfilled the inclusion criteria are reported in Table 1. Other characteristics of patients at hospital and ICU admission are reported in Table S1.

Characteristics of medical treatments and ventilatory management on days 1, 7, and 15 after ICU admission and on the day of tracheostomy are reported in Table S2 and Table S3, respectively.

The median time to tracheostomy was 15 (1–64) days. Seventy-six (49.67%) patients underwent early tracheostomy, while 77 (50.33%) underwent late tracheostomy.

Percutaneous tracheostomy was more frequent than open (surgical) techniques (*n* = 100, 65.36% versus *n* = 53, 34.64%, respectively).

The reasons for tracheostomy included inability to be weaned from the ventilator (*n* = 143, 93.5%), neurological impairment (*n* = 5, 3.3%), extubation failure (*n* = 2, 1.3%), airway failure (*n* = 1, 0.7%), and were unspecified in 2 patients (1.3%). Thirty (19.6%) patients ultimately underwent tracheostomy closure.

Weaning from the ventilator was mainly performed by using the spontaneous breathing trial (SBT) technique in pressure-support ventilation mode (*N* = 84, 54.9%).

The median time from endotracheal intubation to SBT was 12.0 (1.0–35.0) days. The median time from endotracheal intubation to extubation from the endotracheal tube was 12.0 (1.0–28.0) days, whereas the median time from extubation from the endotracheal tube to tracheostomy was 13.0 (1.0–52.0) days.

Nineteen patients were ultimately extubated from the endotracheal tube before tracheostomy, while 133 were never extubated before tracheostomy (missing *N* = 1 patient). Among those who were extubated, 4 (21.1%) underwent early tracheostomy, while 15 (78.9%) underwent late tracheostomy. Among those never extubated from the endotracheal tube before tracheostomy, 71 (53.4%) underwent early tracheostomy, while 62 (46.6%) underwent late tracheostomy (*p* = 0.008). Finally, all the included 153 patients underwent tracheostomy.

After tracheostomy, 85 (55.6%) patients gradually become free of an artificial airway (10 of them died during ICU stay, 1 patient missing data); 49 (32.0%) patients were never extubated from the tracheostomy tube and all of them died during ICU stay, while data of 19 (12.4%) patients were missing.

A higher rate of tracheostomy closure was found in patients who underwent a surgical versus percutaneous tracheostomy (OR 4.23; 95% CI 1.00–17.86, *p* = 0.049).

### 3.1. Cumulative Probability of Survival

**Figure 1:**
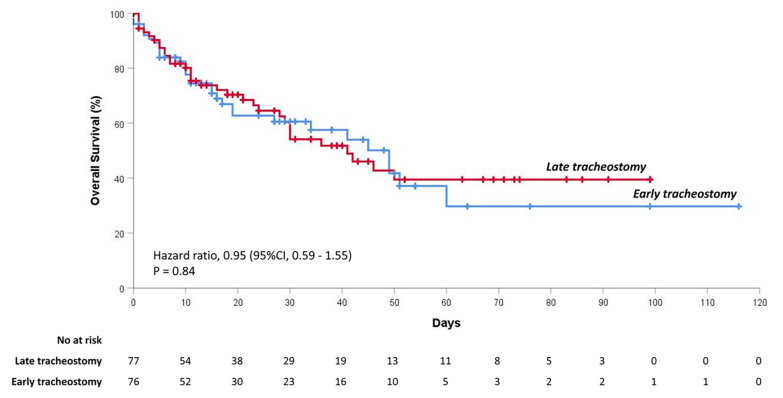
Overall survival of critically ill COVID-19 patients after receiving tracheostomy before or after 15 days. Kaplan–Meier curve of cumulative probability of survival. Patients were grouped by the timing of tracheostomy: early or late (cut-off, 15 days).

The cumulative probability of survival in the overall population is shown in Figure S1. Timing and causes of death are reported in Table S4. The cumulative probability of survival in critically ill COVID-19 patients did not differ between those who received an early tracheostomy and those who were tracheostomized later (*p* = 0.84). Figure 1 shows the cumulative probability of survival after early or late tracheostomy at follow-up (up to 60 days).

**Figure 2:**
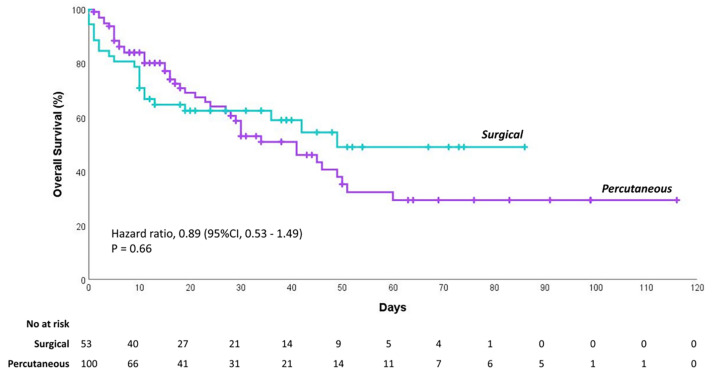
Overall survival of critically ill COVID-19 patients after receiving percutaneous versus surgical tracheostomy. Kaplan–Meier curve of cumulative probability of survival in percutaneous versus surgical tracheostomy groups.

The probability of survival after tracheostomy did not differ significantly between patients who received surgical versus percutaneous tracheostomy (*p* = 0.66), as shown in Figure 2.

The probability of survival did not differ significantly between patients who received early versus late surgical tracheostomy, and early versus late percutaneous tracheostomy (Figures S2 and S3).

### 3.2. ICU Length of Stay

Early tracheostomy resulted in shorter overall ICU stay (time between ICU admission and ICU discharge) when compared to late tracheostomy [median 24.0 (5.0–98.0) versus 46.0 (16.0–133.0) days, *p* < 0.001]. Similarly, percutaneous dilatational tracheostomy resulted in shorter overall ICU stay when compared to surgical tracheostomy [median 30.0 (5.0–133.0) versus 40.0 (20.0–103.0) days, *p* = 0.050].

When considering post-tracheostomy ICU length of stay (from the day of tracheostomy to the day of ICU discharge), the median length of stay for those undergoing early and late tracheostomy was 15.0 (1.0–82.0) days and 22.5 (1.0–86.0) days, respectively (*p* = 0.031).

Again, percutaneous compared to surgical tracheostomy resulted in shorter ICU length of stay [median 16.0 (1.0–82.0) days and 24.0 (6.0–86.0) days, respectively], but not significantly so (*p* = 0.070).

### 3.3. Post-Tracheostomy Complications

Complications after early and late tracheostomy, as well as in surgical and percutaneous tracheostomy groups, are reported in Table 2. The most common pathogens identified in the bronchoalveolar lavage fluid before and after tracheostomy are reported in Table S5.

## 4. Discussion

Few studies comparing early and late and surgical and percutaneous tracheostomy techniques in COVID-19 have been published to date.

In our cohort of tracheostomized critically ill patients with COVID-19 pneumonia, we found that: (1) the median time to tracheostomy was 15 (1–64) days; (2) there was no difference in survival in patients who received tracheostomy before or after 15 days, nor between surgical and percutaneous techniques; (3) early and percutaneous tracheostomy did not improve outcomes, but was associated with shorter ICU stay; (4) post-tracheostomy infection of the lower respiratory tract were less frequent with percutaneous dilatational tracheostomy than with surgical techniques.

### 4.1. Timing and Type of Tracheostomy

Our data on COVID-19 subjects revealed a median time to tracheostomy of 15 (1–64) days. This was set as the cut-off for defining the early and late time of tracheostomy. In fact, 76 (49.67%) patients underwent early tracheostomy, while 77 (50.33%) patients underwent late tracheostomy. Additionally, in our cohort of critically ill COVID-19 patients, percutaneous tracheostomy was performed in 100 patients, while surgical tracheostomy was performed in 53 patients. Of importance, although considered as a secondary endpoint, 19 patients were extubated (all of them reintubated), while 133 were never extubated before tracheostomy. Among those who were extubated, 21.1% underwent early tracheostomy, while 78.9% underwent late tracheostomy (*p* = 0.008). This can be interpreted as the extubation followed by re-intubation could have influenced the time of tracheostomy and ICU length of stay.

Conventionally, tracheostomy is performed in general ICU patients with ongoing mechanical ventilation up to 3 weeks after endotracheal intubation [9], with an increasing tendency to reduce the time to tracheostomy. Studies and meta-analyses investigating the effect of tracheostomy timing on clinical outcomes have reported variable results. In patients with acute respiratory distress syndrome (ARDS), the mean time to tracheostomy is 14 days [20], while 10 days is considered as a conventional cut-off for early versus late tracheostomy in general ICU patients [13].

Several national protocols and international guidelines have published guidance on the management of tracheostomy in COVID-19, despite the scarcity of evidence in the literature [16,21]. During the COVID-19 pandemic, the mean time to tracheostomy has often been longer than usual. Kwak et al. [22] reported a mean time of 12.2 days, very close to that reported in a prospective observational study of 100 tracheostomized COVID-19 patients in the United Kingdom (13.9 ± 4.5 days) [23]. In a cohort of 270 mechanically ventilated patients, of whom 98 underwent percutaneous dilatational tracheostomy, the mean time to tracheostomy was 10.6 ± 5 days [24], shorter than that reported by Tang et al. [25] and Chao et al. [26].

As shown in the COVID-19 literature, the timing of tracheostomy must strike a balance between possible risks to clinicians and optimal outcomes for patients [22,27]. Information on the massive viral load and high infectivity of SARS-CoV-2 appears to have led to delays or even avoidance of tracheostomy in this population. In the early stage of ICU admission (within 7 days after endotracheal intubation), the literature on general non-COVID-19 patients agrees concerning the possible deferment of more aggressive treatment approaches, thus limiting the potential favorable effects of tracheostomy over the possible risk of periprocedural complication [28]. This has been emphasized in COVID-19, since during the early phase of the disease, patients can present with a higher viral load, increasing the risk of infection of health care providers [28]. Several meta-analyses of studies conducted in non-COVID-19 patients have concluded that percutaneous techniques are performed more quickly than surgical tracheostomy [29,30]. This is even more important in the COVID-19 setting, in which the time of operator exposure to the virus takes on a pivotal role; open surgical techniques are presumed to pose a greater risk to the operator, thus requiring more caution.

### 4.2. Cumulative Probability of Survival

In our study, we find no differences in survival in patients who received tracheostomy before or after 15 days. Moreover, survival did not differ significantly between patients who received early versus late surgical tracheostomy, and early versus late percutaneous tracheostomy.

Rosano et al. [18] investigated if early percutaneous tracheostomy (cut-off 10 days) was independently associated with increased hospital mortality. Tracheostomized patients significantly died more than non-tracheostomized. However, this study did not investigate survival between early and late tracheostomies, being of limited interest for our aims. In a smaller report of 29 COVID-19 patients, early tracheostomy did not influence mortality [31]. As reported by Kwak et al. and Shultz et al. [22,32], late tracheostomy did not significantly influence survival in studies that assessed this parameter, which is in line with our findings. As for surgical and percutaneous tracheostomies and survival, no data are available in the literature, making our study the first one assessing this endpoint. Nevertheless, we strongly believe that length of stay is a more interesting outcome to assess in this patient population.

### 4.3. Length of ICU Stay

In our study, early tracheostomy was associated with a shorter overall ICU stay. This should be highlighted since the ICU stay might be considered as a better outcome than survival in COVID-19 patients. It also resulted as being of significance when considering “tracheostomy-ICU stay”, suggesting that the time of tracheostomy might have influenced ICU length of stay. Of interest also was that percutaneous tracheostomy shortened ICU stay significantly as compared to surgical tracheostomy, but it was not confirmed when “tracheostomy-ICU stay” was considered.

In studies including non-COVID-19 patients [6,33,34,35], early tracheostomy was associated with shorter overall ICU stay, shorter duration of sedation, and reduced long-term mortality when compared to late tracheostomy. However, despite its advantages, early tracheostomy in general ICU patients is not clearly supported by the literature [9,10,11,12,13].

In the abovementioned report of 29 COVID-19 patients, early tracheostomy did not influence ICU length of stay [31]. Conversely, the Queen Elizabeth Hospital Birmingham COVID-19 airway team reported a shorter ICU length of stay for early compared to late tracheostomy (cut-off at 14 days), with *p* < 0.0001 [23]. Differently from our study, significance in ICU length of stay was not identified when considering “tracheostomy-ICU stay”. Controversially, Kwak et al. calculated the overall-ICU length of stay (between the day of ICU admission and the day of ICU discharge), missing results on the possible intervention of tracheostomy to this endpoint.

### 4.4. Post-Tracheostomy Complications

In our study, the time to tracheostomy did not significantly influence the rate of complications. Additionally, surgical tracheostomy was associated with significantly lower respiratory tract infections than percutaneous tracheostomy. The pathogens most commonly identified before and after tracheostomy included *Pseudomonas aeruginosa, Klebsiella pneumoniae, Staphylococcus aureus, Candida albicans,* and *Acinetobacter baumannii.*

Massick et al. [36] reported a higher incidence of complications with percutaneous technique than open surgical tracheostomy. Similarly, Silvester et al. [14] reported a higher incidence of bleeding requiring surgical intervention in the percutaneous group, while infections were more frequent in the surgical group. Antonelli et al. [37], in a randomized controlled trial, found higher rates of systemic infectious complications in the surgical group than in the percutaneous groups (more positive pharyngeal swabs and tracheal aspirates and blood cultures), but the difference was not significant. Terragni et al. [11] reported similar rates of ventilator-associated pneumonia between surgically and percutaneously tracheostomized patients. Likewise, Sole et al. [38] reported no significant differences in lower respiratory tract infections before and after dilatational percutaneous tracheostomy (52% pre and 48% post).

Few studies investigated the type and rate of complications in the non-COVID-19 population. The impact of the time of tracheostomy on complications might be considered of great interest in COVID-19, as the time of disease represents a pivotal parameter to understand the hyperinflammatory state and coagulation disorders typical of certain stages of COVID-19 disease [39,40].

Two recent studies reported that in their cohorts of 201 and 100 tracheostomized COVID-19 patients respectively, of whom 124 and 70 underwent percutaneous tracheostomies, while 77 and 30 underwent surgical tracheostomies, no differences in the rate of perioperative complications were found [19,41]. In the smaller of these cohorts [41], 26 (55.3%) patients experienced stoma infection, hemorrhage, and subcutaneous emphysema. Botto et al. reported local infections, hemorrhage, and subcutaneous emphysema in 52.9% after percutaneous tracheostomy, versus 60.0% after surgical tracheostomy. However, complication rates were not significantly different between groups [41]. The pathogens identified in the smaller cohort of 30 COVID-19 patients were slightly different from those identified in our sample [41]. In another study, delayed complications of tracheostomy included cuff leaks and bleeding at the stoma site [42]. Picetti et al. [43], in 66 COVID-19 ICU patients who underwent tracheostomy, reported stoma infection and hemorrhage as the most common complications and the time to tracheostomy as “very early” (around 6 days). Tang et al. reported tracheostoma bleeding as the leading complication [14 (17.5%)], with major bleeding occurring in 4 patients (5.0%) [25], but with a time to tracheostomy performance of 17.5 days. As abovementioned, this may be explained by the variety of clinical presentations of COVID-19 coagulopathy [39,40]. In fact, antithrombotic treatment and the natural history of the disease itself may lead to hemorrhagic or pro-coagulative complications, especially in the early phase, with an increase in pro-inflammatory cytokines, coagulation disorders, and endothelial and microvascular cell damage, that may be variable in timing and presentation [44]. Therefore, the choice to delay tracheostomy in this patient population seems reasonable at face value. However, COVID-19 is often associated with longer ventilator time than expected, more patient-ventilator asynchronies, and a more difficult and longer weaning phase than in non-COVID-19 critical illness. Finally, the fact that prolonged mechanical ventilation is associated with more complications and longer ICU stay suggests that early tracheostomy should be deployed more widely in this patient cohort. Finally, we might conclude that percutaneous tracheostomy seems to cause fewer infectious complications than surgical techniques, both in COVID-19 and non-COVID-19 cohorts, although it is associated with a higher risk of bleeding.

### 4.5. Limitations

Several limitations of the present study must be addressed. First, due to its retrospective nature, some key statistical tests could not be performed, and significant biases may have affected patient selection. Second, temporal relationships are frequently difficult to assess. Third, retrospective studies often need very large sample sizes to detect rare outcomes.**** Even if a patient is ready for ICU discharge, “time of ICU discharge” may not be the same as “time of ICU discharge decision” due to organizational problems. Additionally, no data on general complications were collected (including polyneuropathy or critical illness), but only data concerning complications related to tracheostomy as interest for our primary and secondary endpoints, while a huge amount of data concerning the management of neuromuscular paralysis were missing, as expected in a retrospective study. In fact, most of the centers could not have been retrospectively accessed as to the reason for percutaneous or surgical tracheostomy, therefore potentially influencing outcomes.

## 5. Conclusions

In critically ill COVID-19 patients, the median time to tracheostomy was longer than described in non-COVID-19 subjects. The timing and type of tracheostomy did not affect survival but did shorten overall-ICU stay (shorter for early and percutaneous tracheostomy), even when considering “tracheostomy-ICU stay”. Post-tracheostomy infection of the lower respiratory tract was less frequent with percutaneous dilatational tracheostomy than with the surgical technique. A randomized multicenter trial is warranted and clearly needed to elucidate the potential benefit of early and percutaneous tracheostomy in critically ill, mechanically ventilated COVID-19 patients.

## Figures and Tables

**Table 1:** Overall characteristics at hospital and ICU admission of 153 tracheostomized COVID-19 patients.

Baseline Characteristics	Overall	Time to Tracheostomy			Type of Tracheostomy		
	*n* = 153	Early *n* = 76	Late *n* = 77	*p*-Value	Percutaneous *n* = 100	Surgical *n* = 53	*p*-Value
**Age**, years	63.4 ± 9.34 64.0 (32.0–89.0)	63.8 ± 9.24 65.0 (34.0–89.0)	62.9 ± 9.48 63.0 (32.0–88.0)	0.36	63.9 ± 9.18 64.0 (34.0–88.0)	62.3 ± 9.64 62.0 (32.0–89.0)	0.29
**Sex**, males	118 (77.1)	60 (78.9)	58 (75.3)	0.59	81 (81.0)	37 (69.8)	0.12
**BMI**, kg/m^2^	28.8 ± 5.04 27.8 (16.5–50.8)	29.0 ± 5.19 27.8 16.5 50.8	28.6 ± 4.91 27.8 (19.5–46.4)	0.62	28.7 ± 4.51 27.8 (20.7–46.4)	29.0 ± 5.96 27.8 (16.5–50.8)	0.96
**Comorbidities**							
Hypertension	82 (53.6)	42 (55.3)	40 (51.9)	0.68	57 (57.0)	25 (47.2)	0.25
Diabetes mellitus	34 (22.2)	20 (26.3))	14 (18.2)	0.23	22 (22.0)	12 (22.6)	0.93
Chronic respiratory disease	16 (10.5)	11 (14.7)	5 (6.5)	0.10	13 (13.1)	3 (5.7)	0.15
Chronic cardiac disease	23 (15.0)	12 (15.8)	11 (14.3)	0.80	13 (13.0)	10 (18.9)	0.33
Malignancy	12 (7.8)	5 (6.6)	7 (9.1)	0.56	6 (6.0)	6 (11.3)	0.24
Chronic liver disease	6 (3.9)	3 (3.9)	3 (3.9)	0.99	6 (6.0)	0 (0.0)	0.07
Chronic neurologic disease	13 (8.5)	9 (11.8)	4 (5.2)	0.14	12 (12.0)	1 (1.9)	0.033
Chronic kidney disease	9 (5.9)	5 (6.6)	4 (5.2)	0.72	6 (6.0)	3 (5.7)	0.93
Smoke	10 (7.8)	5 (8.5)	5 (7.1)	0.78	8 (8.3)	2 (6.1)	0.67
**Symptoms’ onset to hospital admission**, days	6.3 ± 5.61 5.0 (0.0–36.0)	6.0 ± 4.85 5.0 (0.0–33.0)	6.6 ± 6.40 5.0 (0.0–36.0)	0.96	6.9 ± 6.26 6.0 (0.0–36.0)	5.2 ± 4.02 4.0 (0.0–19.0)	0.10
**Hospital admission to COVID-19 swab**, days	1.5 ± 5.02 0.0 (0.0–31.0)	1.5 ± 5.74 0.0 (0.0–31.0)	1.5 ± 4.22 0.0 (0.0–31.0)	0.03	0.8 ± 3.04 0.0 (0.0–26.0)	3.0 ± 7.56 0.0 (0.0–31.0)	0.05
**Hospital to ICU admission**, days	6.0 ± 9.12 3.0 (0.0–61.0)	5.8 ± 9.54 3.0 (0.0–61.0)	6.3 ± 8.75 4.0 (0.0–39.0)	0.43	5.4 ± 7.47 3.0 (0.0–39.0)	7.3 ± 11.60 3.0 (0.0–61.0)	0.47

Table legend: data expressed as mean ± standard deviation, median (min-max), or number (percentage) as appropriate. BMI, body mass index; ICU, intensive care unit; COVID-19, coronavirus disease 2019; Early tracheostomy: performed within the first 15 days from endotracheal intubation; Late tracheostomy: performed ≥ 15 days from endotracheal intubation.

**Table 2:** Complications of tracheostomy.

Complications of Tracheostomy	Overall Population	Time to Tracheostomy				Type of Tracheostomy			
		Early	Late	Univariate Analysis *p*-Value OR (95% CI)	Multivariate Analysis *p*-Value OR (95% CI)	Percutaneous	Surgical	Univariate Analysis *p*-Value OR (95% CI)	Multivariate Analysis *p*-Value OR (95% CI)
**Hemorrhage**									
No	128 (90.8)	68 (95.8)	60 (85.7)	0.05	0.09	83 (87.4)	45 (97.8)	0.06	
Yes	13 (9.2)	3 (4.2)	10 (14.3)	3.78 (0.99–14.37)	7.98 (0.72–89.11)	12 (12.6)	1 (2.2)	0.15 (0.02–1.22)	
**Coagulation**									
No	138 (97.9)	69 (97.2)	69 (98.6)	0.99		92 (96.8)	46 (100.0)	0.55	
Yes	3 (2.1)	2 (2.8)	1 (1.4)			3 (3.2)	0 (0.0)		
**Tracheal stenosis**									
No	139 (100.0)	71 (100.0)	68 (100.0)	/		93 (100.0)	46 (100.0)	/	
Yes	0 (0.0)	0 (0.0)	0 (0.0)			0 (0.0)	0 (0.0)		
**Infection of stoma**									
No	128 (90.8)	64 (90.1)	64 (91.4)	0.79		84 (87.5)	44 (97.8)	0.06	
Yes	13 (9.2)	7 (9.9)	6 (8.6)			12 (12.5)	1 (2.2)	0.16 (0.02–1.26)	
**Pneumothorax**									
No	137 (100.0)	66 (95.7)	66 (94.3)	0.71		86 (92.5)	46 (100.0)	0.10	
Yes	7 (5.0)	3 (4.3)	4 (5.7)			7 (7.5)	0 (0.0)		
LRTI *									
Negative	30 (34.5)	7 (22.6)	23 (41.1)	0.09	0.07	24 (46.2)	6 (17.1)	0.06	0.007
Positive	57 (65.5)	24 (77.4)	33 (58.9)	0.42 (0.16–1.13)	0.27 (0.07–1.11)	28 (53.8)	29 (82.9)	4.14 (1.47–11.66)	10.06 (1.89–53.54)

Table Legend: data expressed as number (percentage). Multivariate analysis adjusted for age, sex, comorbidities, body mass index, smoking, and type of tracheostomy. Abbreviations: LRTI, lower respiratory tract infection; OR odds ratio (late vs. early; and surgical vs. percutaneous); CI, confidence interval. * LRTI diagnosed (with positive bronchoalveolar lavage fluid) only after tracheostomy, with no evidence of infection before.

## Data Availability

The data presented in this study are available on reasonable request from the corresponding author.

## Supplementary Materials

The following are available online at https://www.mdpi.com/article/10.3390/jcm10122651/s1: List of participating centers [p. 2]. Table S1. Baseline characteristics of patients at ICU admission stratified for timing and type of tracheostomy. [p. 3, 4]. Table S2. Treatment characteristics of COVID-19 patients. [p. 5]. Table S3. Characteristics of ventilatory management on days 1, 7, 15 from ICU admission until the day of tracheostomy. [p. 6]. Figure S1. Cumulative survival of critically ill COVID-19 patients after tracheostomy. [p. 7] Table S4. Timing and cause of death COVID-19 patients. [p. 8]. Figure S2. Cumulative survival after receiving percutaneous tracheostomy (early vs. late) [p. 9]. Figure S3. Cumulative survival after receiving surgical tracheostomy (early vs. late) [p. 9]. Table S5. Pathogens identified in bronchoalveolar lavage fluid before and after tracheostomy. [p. 10].
