# Supplementary material for: Tracheostomy Timing and Outcome in Severe COVID-19: The WeanTrach Multicenter Study
Source: J Clin Med. 2021 Jun 16;10(12):2651. doi: 10.3390/jcm10122651 (PMC8235219; doi:10.3390/jcm10122651)
Supplement: Supplementary file 1 [file jcm-10-02651-s001.zip › jcm-1222263-supplementary.pdf]

Supplemental Material (SM)

Tracheostomy timing and outcome: the WeanTrach multicenter study

<u>**SM contents:**</u>

List of participating centers [p. 2].

Table S1. Baseline characteristics of patients at ICU admission stratified for timing and type of tracheostomy. [p. 3, 4]

Table S2. Treatment characteristics of COVID-19 patients. [p. 5].

Table S3. Characteristics of ventilatory management on days 1, 7, 15 from ICU admission until the day of tracheostomy. [p. 6].

Figure S1. Cumulative survival of critically ill COVID-19 patients after tracheostomy. [p. 7]

Table S4. Timing and cause of death COVID-19 patients. [p. 8]

Figure S2. Cumulative survival after receiving percutaneous tracheostomy (early vs late) [p. 9]

Figure S3. Cumulative survival after receiving surgical tracheostomy (early vs late) [p. 9]

Table S5. Pathogens identified in bronchoalveolar lavage fluid before and after tracheostomy. [p. 10]

List of participating centers

Anesthesia and Intensive Care, Policlinico San Martino Hospital, IRCCS for Oncology and Neuroscience, Genoa, Italy (coordinating center); Department of Otorhinolaryngology-Head and Neck Surgery, University of Genoa, San Martino Policlinico Hospital IRCCS for Oncology and Neuroscience, Genoa, Italy; Department of Otorhinolaryngology-Head and Neck Surgery, University of Brescia, ASST-Spedali Civili of Brescia, Brescia, Italy; First Division of Anesthesiology and Intensive Care Unit, ASST Spedali Civili di Brescia, 25123, Brescia Italy; Dipartimento dell'Emergenza e Trapianti d'Organo (DETO), Sezione di Anestesiologia e Rianimazione, Università degli Studi di Bari "Aldo Moro", Ospedale Policlinico, Bari, Italy; Anaesthesia and Intensive Care Department, University Hospital, Ospedale di Circolo, Varese, Italy; Anestesia e Rianimazione 1U, Department of Anesthesia, Intensive Care and Emergency, Città della Salute e della Scienza Hospital, Turin, Italy; Department of Anaesthesia, Intensive Care and Pain Management, Universita Campus Bio-Medico di Roma, via Alvaro del Portillo 21, Rome, Italy; U.O. di Anestesia e Rianimazione, Ospedale di Treviglio-Caravaggio, Treviglio, Italy; Department of Morphology, Surgery and Experimental medicine, Faculty of Medicine, University of Ferrara, Ferrara, Italy; Anestesia e Rianimazione, Ospedale Villa Scassi, Genoa, Italy; Anesthesia and Intensive Care, "Garibaldi Centro" Hospital, ARNAS Garibaldi, Catania, Italy; Dipartimento di Neuroscienze, Scienze Riproduttive e Odontostomatologiche, Università degli Studi di Napoli Federico II, Napoli, Italy.

Table S1. Baseline characteristics of patients at ICU admission stratified for timing and type of tracheostomy.

Baseline characteristics	Overall	Time to tracheostomy			Type of tracheostomy		
	N = 153	Early<br>N = 76	Late<br>N = 77	P value	Percutaneous<br>N = 100	Surgical<br>N = 53	P value
**Patients directly admitted to ICU**	28 (18.3)	14 (18.4)	14 (18.2)		21 (21.0)	7 (13.2)	
**Endotracheal intubation**				0.034			0.08
Before ICU admission	18 (11.8)	4 (5.3)	14 (18.2)		8 (8.0)	10 (18.9)	
On the same day	124 (81.0)	67 (88.2)	57 (74.0)		86 (86.0)	38 (71.7)	
During ICU stay	11 (7.2)	5 (6.6)	6 (7.8)		6 (6.0)	5 (9.4)	
**ICU admission to endotracheal intubation, days**	N = 135<br>0.6 ± 3.18<br>0.0 (0.0 – 31.0)	N = 72<br>0.9 ± 4.19<br>0.0 (0.0-31.0)	N = 63<br>0.3 ± 1.16<br>0.0 (0.0-7.0)	0.64	N = 92<br>0.7 ± 3.59<br>0.0 (0.0-31.0)	N = 43<br>0.6 ± 2.05<br>0.0 (0.0-10.0)	0.59
**Type of respiratory support at ICU admission**				0.09			0.22
COT (mild)	12 (7.8)	5 (6.6)	7 (9.1)		6 (6.0)	6 (11.3)	
NIMV (moderate)	67 (43.8)	40 (52.6)	27 (35.1)		41 (41.0)	26 (49.1)	
IMV (severe)	74 (48.4)	31 (40.8)	43 (55.8)		53 (53.0)	21 (39.6)	
**Prone position**	93/151 (61.6)	43/74 (58.1)	50/77 (64.9)	0.39	65/98 (66.3)	28/53 (52.8)	0.10
**Ventilation type**				-			-
COT (mild)	1 (0.7)	1 (1.3)	0 (0.0)		0 (0.0)	1 (1.9)	
NIMV (moderate)	1 (0.7)	1 (1.3)	0 (0.0)		0 (0.0)	1 (1.9)	
IMV (PCV)	57 (37.3)	17 (22.4)	40 (51.9)		36 (36.0)	21 (39.6)	
IMV (VCV)	46 (30.1)	19 (25.0)	27 (35.1)		22 (22.0)	24 (45.3)	
IMV (AV)	5 (3.3)	2 (2.6)	3 (3.9)		3 (3.0)	2 (3.8)	
Not reported	43 (28.1)	36 (47.4)	7 (9.1)		39 (39.0)	4 (7.5)	
**Blood gas analysis**							
PaO_2/FiO_2	N = 107<br>155.4 ± 74.40<br>138.3 (41.7-357.5)	N = 38<br>134.1 ± 55.39<br>125.4 (53.0-323.3)	N = 69<br>167.2 ± 81.02<br>148.8 (41.7-357.5)	0.05	N = 58<br>152.8 ± 70.05<br>140.4 (41.7-357.5)	N = 49<br>158.5 ± 79.86<br>130.0 (51.1-350.0)	0.93

$PaCO_2$, mmHg	N = 107<br>46.5 ± 11.98<br>45.0 (22.0-100.0)	N = 38<br>46.7 ± 12.24<br>46.5 (22.0-100.0)	N = 69<br>46.3 ± 11.92<br>44.0 (28.0-83.8)	0.56	N = 58<br>48.1 ± 13.43<br>45.6 (28.0-100.0)	N = 49<br>44.6 ± 9.80<br>44.0 (22.0-83.0)	0.27
**IMV setting**							
Minute volume, L/min	N = 106<br>9.0 ± 1.99<br>8.5 (2.8-15.0)	N = 37<br>8.7 ± 2.04<br>8.4 (2.8-14.3)	N = 69<br>9.1 ± 1.97<br>8.5 (6.0-15.0)	0.62	N = 59<br>8.7 ± 1.96<br>8.4 (2.8-14.4)	N = 47<br>9.4 ± 1.99<br>9.0 (5.6-15.0)	0.07
PEEP, cmH_2O	N = 108<br>12.0 ± 3.00<br>12.0 (5.0-20.0)	N = 38<br>11.5 ± 2.90<br>12.0 (5.0-17.0)	N = 70<br>12.3 ± 3.03<br>12.0 (6.0-20.0)	0.26	N = 61<br>11.4 ± 2.81<br>12.0 (5.0-18.0)	N = 47<br>12.7 ± 3.09<br>13.0 (7.0-20.0)	0.017
Tidal volume, ml/kg	N = 107<br>5.8 ± 1.06<br>5.9 (3.3-9.2)	N = 38<br>5.7 ± .87<br>5.8 (4.1-7.9)	N = 69<br>5.9 ± 1.16<br>6.0 (3.3-9.2)	0.40	N = 60<br>5.7 ± 1.06<br>5.7 (3.3-9.2)	N = 47<br>6.0 ± 1.07<br>6.0 (4.1-8.3)	0.13

Table Legend: ICU, intensive Care Unit; IMV, invasive mechanical ventilation; COT, conventional oxygen therapy; NIMV, non-invasive mechanical ventilation; PCV, pressure-controlled ventilation; VCV, volume-controlled ventilation; AV, assisted ventilation; PaO_2, arterial partial pressure of oxygen; FiO2, fraction of inspired oxygen; $PaCO_2$, partial pressure of carbon dioxide; PEEP, positive end-expiratory pressure. Data expressed as absolute number (percentage), mean ± standard deviation, or median (IQR) as appropriate. IQR: interquartile range.

Table S2. Treatment characteristics of critically ill COVID-19 patients.

		Treatment characteristics of COVID-19 patients (N = 153)
**Corticosteroids**		
	No	32 (20.9)
	Yes	121 (79.1)
**Tocilizumab**		
	No	104 (69.3)
	Yes	46 (30.7)
**Immunoglobulins**		
	No	141 (93.4)
	Yes	10 (6.6)
**Antivirals**		
	No	33 (22.1)
	Yes	116 (77.9)
Antivirals used per patient, number		1.6 ±1.2<br>2.0 (0.0 - 5.0)
Type of antiviral		
Remdesivir		
	No	132 (88.6)
	Yes	17 (11.4)
Ritonavir		
	No	99 (66.4)
	Yes	50 (33.6)
Cobicistat		
	No	114 (76.5)
	Yes	35 (23.5)
Darunavir		
	No	85 (57.0)
	Yes	64 (43.0)
Hydroxychloroquine		
	No	129 (86.6)
	Yes	20 (13.4)
Lopinavir		
	No	111 (74.5)
	Yes	38 (25.5)
Oseltamivir		
	No	137 (91.9)
	Yes	12 (8.1)

Table Legend: Data expressed as absolute number (percentage), mean ± standard deviation, or median (IQR) as appropriate. IQR: interquartile range.

Table S3. Characteristics of ventilatory management on days 1, 7, 15 from ICU admission until the day of tracheostomy.

Ventilatory parameter	Day 1	Day 7	Day 15	Day of tracheostomy*
**Ventilation type**				
COT (mild)	1 (0.7)	9 (5.9)	2 (1.3)	0 (0.0)
NIMV (moderate)	1 (0.7)	4 (2.6)	2 (1.3)	0 (0.0)
IMV (severe)				
PCV	57 (37.3)	32 (20.9)	47 (30.7)	34 (22.2)
VCV	46 (30.1)	19 (12.4)	22 (14.4)	21 (13.7)
AV	5 (3.3)	43 (28.1)	37 (24.2)	52 (34.0)
Not reported	43 (28.1)	46 (30.1)	43 (28.1)	42 (27.5)
**Blood gas analysis**				
PaO_2/FiO_2	N = 107	N = 109	N = 106	N = 107
	138.3 (41.7 - 357.5)	162.0 (58.0 - 460.0)	164.5 (56.0 - 500.0)	168.0 (57.5 - 496.0)
$PaCO_2$, mmHg	N = 107	N = 109	N = 106	N = 106
	45.0 (22.0 - 100.0)	47.0 (27.0 - 137.0)	44.0 (29.0 - 79.0)	46.5 (30.0 - 72.0)
**IMV setting**				
MV, L/min	N = 106	N = 89	N = 68	N = 71
	8.5 (2.8 - 15.0)	9.0 (2.0 -18.0)	9.5 (2.3 - 18.0)	9.4 (1.5 - 15.9)
PEEP, cmH_2O	N = 108	N = 106	N = 97	N = 107
	12.0 (5.0 - 20.0)	10.0 (4.0 - 18.0)	10.0 (4.0 - 15.0)	10.0 (2.0 - 31.0)
Tidal volume, ml/kg	N = 107	N = 88	N = 68	N = 71
	5.9 (3.3 - 9.2)	5.9 (1.5 - 9.2)	5.9 (1.8 - 12.5)	5.8 (2.6 - 12.5)

Table Legend: ICU, intensive care unit; FiO_2, fraction of inspired oxygen; PaO_2, partial pressure of arterial oxygen; $PaCO_2$, partial pressure of arterial carbon dioxide; COT, conventional oxygen therapy; NIMV, non-invasive mechanical ventilation; IMV, invasive mechanical ventilation; VCV, volume-controlled ventilation; PCV, pressure-controlled ventilation; AV, assisted ventilation; PEEP, positive end-expiratory pressure; MV, minute ventilation. Data are reported as median (IQR); number of patients (percentage) as appropriate. * Day when tracheostomy performed.

Figure S1. Cumulative survival after tracheostomy in critically ill COVID-19 patients.

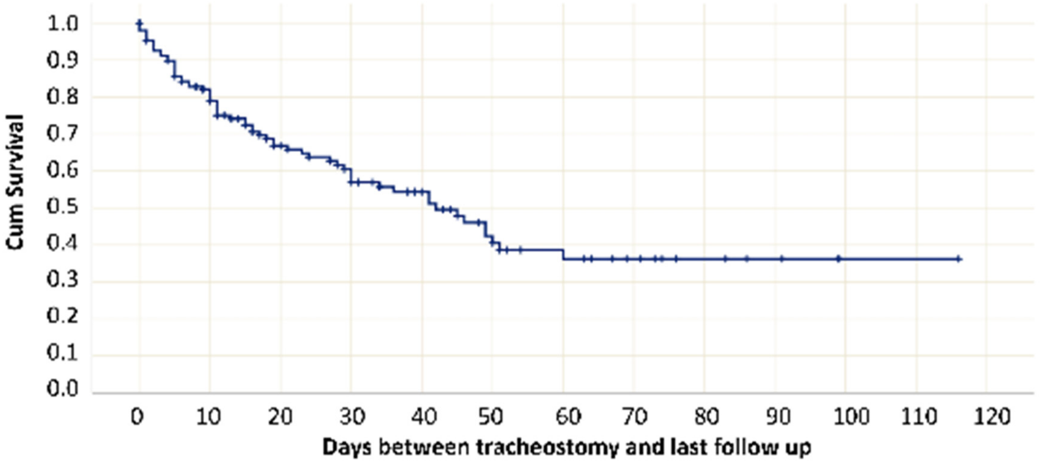

Days of Follow Up	Events	Cum. prop. surv. (S.E.)
10	30	0.790 (0.034)
20	44	0.668 (0.042)
30	53	0.570 (0.047)
40	55	0.544 (0.048)
50	63	0.405 (0.056)
60	65	0.362 (0.058)

Table S4. Timing and cause of death in COVID-19 patients.

	COVID-19 (N = 153)
**Death**	65 (42.5)
**Days elapsed from tracheostomy to death**	25.4 ± 24.47
	16.0 (0.0 – 116.0)
**Cause of death**	
Cardiocirculatory arrest	10 (15.4)
Respiratory failure	6 (9.2)
Multiorgan dysfunction	45 (69.2)
Pulmonary embolism	2 (3.1)
Pancreatitis	1 (1.5)
Not available	1 (1.5)

Table Legend: ICU, intensive care unit. Data expressed as mean ± standard deviation, median (range), or number (percentage) as appropriate.

Figure S2. Cumulative survival after receiving percutaneous tracheostomy (early vs late)

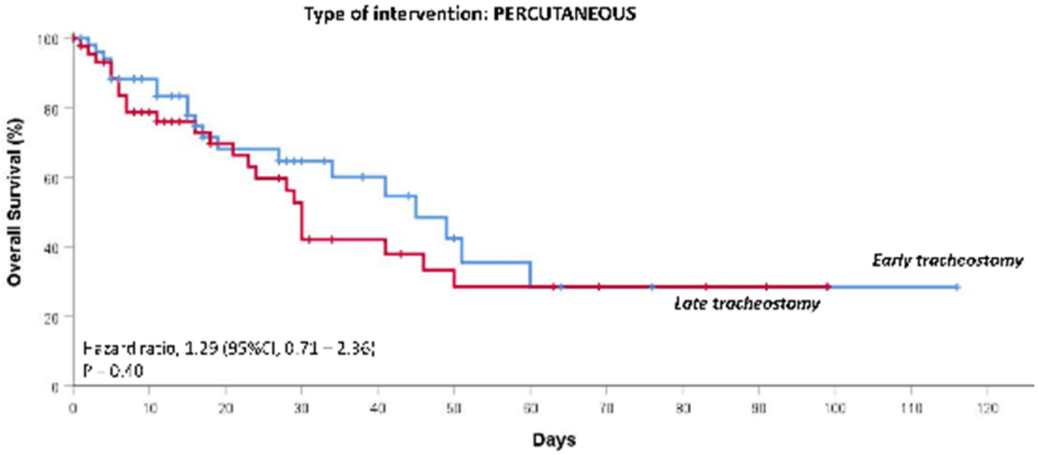

No at risk													
Late tracheostomy	47	30	21	15	10	7	6	4	4	3	3	0	0
Early tracheostomy	53	36	20	15	11	7	5	3	2	2	1	1	0

Figure S3. Cumulative survival after receiving surgical tracheostomy (early vs late)

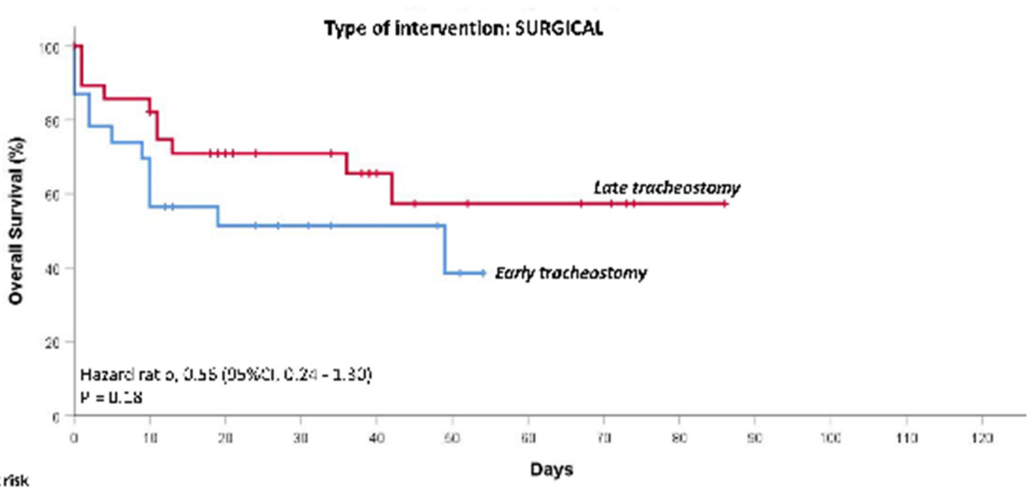

No at risk													
Late tracheostomy	30	24	17	14	9	6	5	4	1	0	0	0	0
Early tracheostomy	23	16	10	7	5	3	0	0	0	0	0	0	0

Table S5. Pathogens identified in bronchoalveolar lavage fluid before and after tracheostomy.

**Pathogens in BALF**	**Pre-tracheostomy**	**Post-tracheostomy**
Patients with ≥ 2 pathogens in BALF	18 (11.8)	30 (19.6)
Patients without available BALF	84 (54.9)	66 (43.1)
Patients with negative BALF	19 (12.4)	17 (11.1)
Patients with positive BALF	50 (32.7)	70 (45.8)
Overall identified pathogens in BALF	75 (100)	107 (100)
Type of pathogens identified in positive BALF	**Pre-tracheostomy**	**Post- tracheostomy**
Pseudomonas aeruginosa	23 (30.7)	29 (27.1)
Klebsiella pneumoniae	10 (13.3)	13 (12.1)
Staphylococcus aureus	7 (9.3)	9 (8.4)
Candida albicans	6 (8.0)	7 (6.5)
Acinetobacter baumannii	5 (6.7)	9 (8.4)
Stenotrophomonas maltophilia	4 (5.3)	4 (3.7)
Candida parapsilosis	3 (4.0)	3 (2.8)
Aspergillus fumigatus	1 (1.3)	7 (6.5)
Others	*C. glabrata, C. tropicalis, P. mirabilis, A. pittii, A. fumigatus, E. aerogenes, E. cloacae, E. coli, H. influenzae, O. anthropi, P. fluorescens, R. ornithinolytica, S. anginosus, S. hominis, S. capitis*	*C. glabrata, C. tropicalis, C. auris, A. niger, A. pittii, E. aerogenes, E. faecalis, E. faecium, E. cloacae, E. coli, H. influenzae, O. anthropi, K. oxytoca, M. morganii, S. epidermidis, P. putida*

Table Legend: BALF, bronchoalveolar lavage fluid. Data expressed as number (percentage).
